# The role of p.Val444Ala variant in the *ABCB11* gene and susceptibility to biliary atresia in Vietnamese patients

**DOI:** 10.1097/MD.0000000000028011

**Published:** 2021-11-24

**Authors:** Nguyen Van Tung, Nguyen Thi Kim Lien, Nguyen Ngoc Lan, Nguyen Thi Phuong Mai, Pham Thi Hai Yen, Nguyen Pham Anh Hoa, Nguyen Huy Hoang

**Affiliations:** a Institute of Genome Research, Vietnam Academy of Science and Technology, Vietnam; b Graduate University of Science and Technology, Vietnam Academy of Science and Technology, Vietnam; c Vietnam National Children's Hospital, Ministry of Health, Vietnam.

**Keywords:** *ABCB11* gene, biliary atresia, p.Val444Ala variant, the risk factor, Vietnamese patients

## Abstract

Biliary atresia (BA) is the most serious type of obstructive cholangiopathy that occurs in infants. BA can be the cause of death in children under 2 years if untreated early. However, the etiology of the disease is not known. BA is considered to be the result of the destruction of the bile duct system including the accumulation of bile acids. The bile salt export pump, a transporter protein encoded by the *ABCB11* gene, plays the main role in the exportation and accumulation of bile acids. The p.Val444Ala variant in this gene is known to be associated with many cholestatic diseases. However, to date no study have been performed to evaluate the association of this variant with susceptibility to the risk of BA. In this study, we aimed to identify the frequency of p.Val444Ala variant and the risk of BA in Vietnamese patients.

The polymerase chain reaction (PCR)– restriction fragment length polymorphism method was used to determine the frequency of alleles c.1331T>C (p.Val444Ala, rs2287622) in the *ABCB11* gene in 266 Vietnamese patients with BA and 150 healthy people. The gene segment containing the variant was amplified by PCR with specific primers, after that the PCR products were cut by *Hae*III restriction enzyme and analyzed on agarose gel to determine the genotypes. The frequency of alleles was assessed statistically to determine the association between these alleles and the risk of disease in patients.

In our study, the frequency of alleles c.1331T>C (p.Val444Ala, rs2287622) in the *ABCB11* gene was investigated the first time in the patients with BA. The results showed that CC and TC genotypes were significantly different between BA patients and healthy people (*P* < .01), and the C allele was associated with an increased risk of BA (odds ratio = 2.47; 95% confidence interval: 1.84–3.32; *P* < .01). The initial results of clinical, biochemical, and genetic analysis in our study suggested that the p.Val444Ala variant in the *ABCB11* gene may be a susceptibility factor for the disease in Vietnamese patients with BA. These results provided new insights into the role of this *ABCB11* variant in the pathogenesis of BA.

## 1 Introduction

Biliary atresia (BA) is one of the neonatal cholestatic diseases due to biliary fibrosis and obstruction of the intra- and extra-hepatic biliary tract, which reaches up to 1/5000 Asian infants but only 1/18,000 Caucasian infants.^[1]^ If undiagnosed and untreated, BA can lead to death within the first 2 years of age. However, diagnosis of BA is a great challenge and early diagnosis will limit the progression to cirrhosis.^[2]^ For infants with BA, if detected in the first 3 months of life, the Kasai hepatoportoenterostomy (HPE) is performed to restore bile flow and half of the children have the chances of survival after 2 years of age.^[3]^ In many patients, the origination of failure in Kasai HPE is not clear, it might be due to progression of intrahepatic bile duct damages and decline repair of bile duct epithelial cells. Also, to prolong life, 70% to 80% of BA patients need liver transplantation.^[1]^

The etiology and pathogenic mechanism of the BA have not been fully understood. BA is a multi-cause disease, in which, the genetic cause plays a certain role in the formation of the disease.^[4,5]^ To date, many genes have been identified in association with BA.^[6–10]^ Besides that, variants in some genes have also been identified to increase the risk of BA.^[11–17]^ BA is also considered to be a result of several pathogenic processes leading to ascending obstruction of bile ducts which affects both the intrahepatic and extrahepatic biliary duct system.^[18]^ The destruction and rapid progression of biliary fibrosis, possibly due to incessant cholestasis and retention of bile components including bile acids.^[2]^ The bile salt export pump (BSEP), a transporter protein encoded by the *ABCB11* gene, plays the main role in the exportation of the bile acids from the hepatocyte to the bile ducts. Defects in BSEP synthesis and/or function lead to reduced bile salt secretion and accumulation of bile salts. Based on the existing knowledge, a few studies have worked on the potential role of plasma bile acid levels and the contribution of variant p.Val444Ala (c.1331T>C, rs2287622) of *ABCB11* to susceptibility for intrahepatic cholestasis of pregnancy and progressive familial intrahepatic cholestasis (PFIC).^[19–21]^ Sangkhathat et al^[22]^ performed whole exome sequencing analysis in 20 BA patients and identified 13 rare variants in 9 genes that are associated with the disease: 4 in *JAG1* (Alagille syndrome), 2 in *MYO5B* (PFIC type 6), and one each in *ABCC2* (Dubin–Johnson syndrome), *ABCB11* (PFIC type 2), *UG1A1* (Crigler–Najjar syndrome), *MLL2* (Kabuki syndrome), *RFX6* (Mitchell–Riley syndrome), *ERCC4* (Fanconi anemia), and *KCNH1* (Zimmermann–Laband syndrome).

In Vietnam, BA has been reported to be as high as 1 in every 2400 live births.^[23]^ Patients with BA frequently face episodes of biliary tract infections, absorption disorders, delay in physical development, cirrhosis, portal hypertension, and gastrointestinal bleeding. Affected children have irreversible cirrhosis and require liver transplant surgery. Therefore, the study aimed to investigate the potential role of variant p.Val444Ala (c.1331T>C, rs2287622) in the *ABCB11* gene in the risk of disease in Vietnamese patients with BA.

## 2 Materials and methods

### 2.1 Study subjects

**Figure 1:**
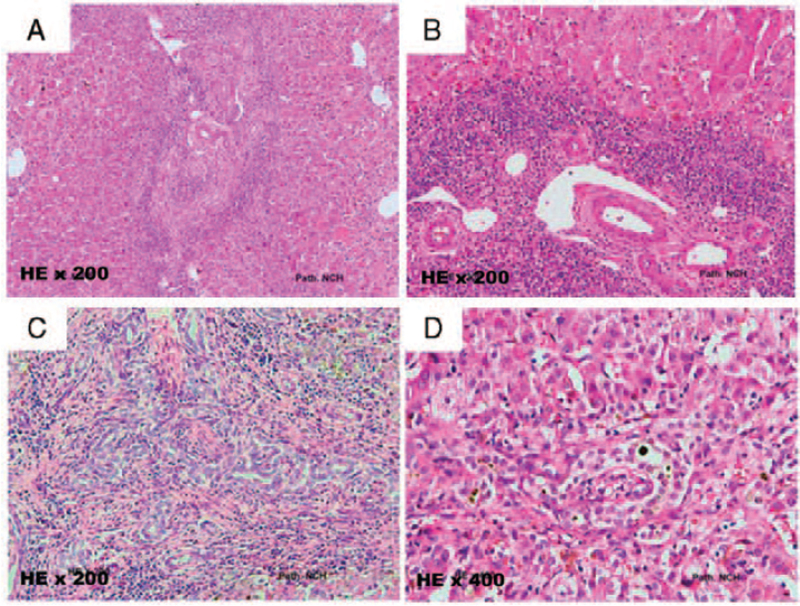
Liver biopsy specimen of some patients with BA in this study. These cases showed typical histopathologic features of biliary atresia. **A.** Liver biopsy specimen from a 78 day-olds with BA shows ductal plate malformation with numerous curvilinear around a central vascular core (H + E, ×200). **B**. Liver biopsy specimen from a 65 day-olds with BA expanded portal tract with severe inflammation around portal tract (H + E, ×200). **C**. Photomicrograph from 68 day-olds with showing the severe grade of bile ductal proliferation (H + E, ×200). **D.** The hepatocellular swelling and giant cell in the liver, cholestasis in hepatocellular and bile ducts in this 60 day-olds with BA (H + E, ×400).

Deoxyribonucleic acid (DNA) was collected from 266 Vietnamese patients who were diagnosed with BA by ultrasonography, biochemical liver function tests, and liver biopsy (Table 1, Fig. 1). The patients underwent Kasai surgery at the age of 2 to 3 months at the Hepatology Department, Vietnam National Children's Hospital. A control cohort of 150 healthy infants (125 boys and 125 girls aged 2–3 months) was formed.

**Table 1:** Clinical characteristics of BA patients in this study.

Clinical characteristics	Patient	Control
Number of patients	266	150
Age (mo)	2.70 ± 1.25	2.64 ± 0.78
Sex (male/female)	126/140	125/125
Type 1 (n)	5	
Type 2 (n)	1	
Type 3 (n)	260	
Direct bilirubin (mg/dL)	5.68 ± 1.69	6.30 ± 1.21
Protein (g/L)	55.92 ± 4.42	58.23 ± 5.06
Albumin (g/L)	36.42 ± 7.87	38.05 ± 3.10
Aspartate aminotransferase (AST) (U/L)	217.43 ± 135.71	240.99 ± 162.85
Alanine aminotransferase (ALT) (U/L)	129.32 ± 88.02	141.02 ± 83.53
Gamma glutamyltransferase (GGT) (IU/L)	604.25 ± 467.81	606.02 ± 440.84
Alkaline phosphatase (ALP) (U/L)	579.34 ± 306.14	744.52 ± 224.90

### 2.2 Ethics statement

The present study protocol was reviewed and approved by the Institutional Review Board of the Institute of Genome Research, Vietnam Academy of Science and Technology (approval No. 06/QD-NCHG). Informed consent was submitted by all subjects when they were enrolled.

### 2.3 DNA extraction and genotyping

DNA extraction was performed using the Gene JET Whole Blood Genomic DNA Purification Mini Kit (Thermo Fisher Scientific, Waltham, MA) according to the manufacturer's instructions. For genotyping of the variant p.Val444Ala (c.1331T>C, rs2287622) in the ATP-binding cassette, subfamily B (MDR/TAP), member 11 *ABCB11* gene, we used the primers included 5′–CACACAGACACCGAGTATCAACACA–3′ as F primer and 5′–CAGGACAGTCTCAATGTATGCTACACCT–3′ as R primer.^[24]^ Amplification was carried out using Dream Taq DNA Polymerase (Thermo Fisher Scientific, Waltham, MA). The initial denaturation temperature was 95°C for 4 minutes followed by cycling, including denaturation at 95°C for 30 seconds, annealing at 58°C for 30 seconds, and extension at 72°C for 60 seconds, for 30 cycles, and a final extension at 72°C for 5 minutes. A polymerase chain reaction (PCR) product of 333 bp contains a cut-off point of *Hae*III restriction enzyme was obtained and digested into 2 fragments of different sizes with *Hae*III restriction enzyme. The C allele was digested to produce bands of 206 and 127 bp in positive samples. While the T allele was not digested in positive samples. And the heterozygous samples formed 3 bands of 333, 206, and 127 bp after digested with *Hae*III restriction enzyme.

### 2.4 Statistical analysis

Data were analyzed using the statistical package SPSS version 23 (IBM, New York). Allele distribution of the variant follows Hardy–Weinberg equilibrium was tested using Chi-squared test (χ^2^). Three models (additive, dominant, recessive) were tested for association of the variant p.Val444Ala (c.1331T>C, rs2287622) with susceptibility to the disease. The odds ratio (OR) and 95% confidence interval (CI) were calculated to detect the risk ratio between patients and controls. A 2-tailed test was used for all of the statistical analyses and *P*-values <.05 were considered statistically significant.

## 3 Results

In this study, 266 patients were diagnosed with BA. According to the classification of the Japanese Association of Pediatric Surgeons, there were 5 patients with type 1 (obstruction at the level of the common bile duct), 1 patient with type 2 (obstruction at the level of the common hepatic duct), and 260 patients with type 3 (obstruction at the level of porta). Type 3 is the commonest and has the worst prognosis. Genotyping of *ABCB11* variant p.Val444Ala was performed in 266 patients with BA and was compared with 150 healthy control in determining allele and genotype frequencies. Table 2 showed that the genotype distribution of variants in the population was in accordance with Hardy–Weinberg equilibrium (*P* > *.05*). The C allele frequency of *ABCB11* variant p.Val444Ala in the patient group was higher than that in the healthy group, suggesting a relation between this allele and disease susceptibility.

**Table 2:** Genotype and allele frequency of variant c.1331T>C (rs2287622) in *ABCB11* gene.

	Genotype			Allele frequency (%)			
	TT	TC	CC	T	C	HWE *P*-value	HWE
Patients (n = 266)	56	130	80	45.5	54.5	.81	+
Control (n = 150)	72	58	20	67.0	33.0	.14	+
Total	128	188	100	55.0	45.0	.06	+

HWE = Hardy–Weinberg equilibrium was checked by Chi-squered test.

Three models of alleles (additive, dominant, and recessive) were performed in statistical analysis. Genotype analysis presented in Table 3 demonstrates that the occurrence of the C allele was significantly over-represented in BA patients (54.51%) than that in the control group (32.67%) (OR = 2.47, 95% CI = 1.84–3.32, *P* *<* *.01*). In the additive model, a significant difference of genotypes was obtained in both the patient group and the control group with *P* *<* *0.01*. The frequency of CC genotype differed significantly between the 2 groups and was associated with an increased risk of BA (OR = 5.14, 95% CI = 2.82–9.39, *P* *<* *.01*) compared to the TT genotype. The frequency of TC genotype also differed significantly between the 2 groups (OR = 2.88, 95% CI = 1.81–4.59, *P* *<* *.01*) compared to the TT genotype. Similarly, the CC genotype was linked to an increased BA risk when compared to the genotype TT + TC (OR = 2.79, 95% CI = 1.63–4.79, *P* *<* *.01*) in the recessive model. Moreover, in the dominant model the frequency of combined TC + CC genotypes in the BA patient group (78.95%) was significantly higher than that in the control group (52.00%) (OR = 3.46, 95% CI = 2.24–5.35, *P* *<* *.01*).

**Table 3:** Association of variant c.1331T>C (rs2287622) in *ABCB11* gene with BA.

Genotype	Patients (n = 266)	Control (n = 150)	OR	95% CI	*P*-value
Additive					
TT	56 (21.05%)	72 (48.00%)	1.00		
TC	130 (48.87%)	58 (38.67%)	2.88	1.81–4.59	<0.01
CC	80 (30.08%)	20 (13.33%)	5.14	2.82–9.39	<0.01
Dominant					
TT	56 (21.05%)	72 (48.00%)	1.00		
TC + CC	210 (78.95%)	78 (52.00%)	3.46	2.24–5.35	<0.01
Recesssive					
TT + TC	186 (69.92%)	130 (86.67%)	1.00		
CC	80 (30.08%)	20 (13.33%)	2.79	1.63–4.79	<0.01
Alleles					
T	242 (45.49%)	202 (67.33%)	1.00		
C	290 (54.51%)	98 (32.67%)	2.47	1.84–3.32	<0.01

95% CI = 95% confidence interval of odds ratio, OR = Odds ratio, *P*-value = calculated by either Fisher exact test or Chi-squared test. *P* < .05 indicates statistical significance.

## 4 Discussion

BA is an inflammatory biliary disease in a newborn, characterized by fibrosis and obstruction of intrahepatic and extrahepatic bile ducts. The destruction of the inner and outer bile ducts, the result of unexplained inflammatory processes, leading to fibrosis, progressive biliary obstruction, and cirrhosis of the liver. The destruction and rapid progression of biliary fibrosis, possibly due to incessant cholestasis and retention of bile components including bile acids.^[25]^ Studies based on fibrogenesis and inflammation suggested that these processes contribute to the formation mechanism of the disease and in certain populations, cholestasis may affect the effectiveness of HPE surgery and subsequent restoration of biliary tract function. And the relationship between BA and other cholestatic diseases has been observed.^[26,27]^

Besides, the genetic participates in the adaptation and response to cholangiopathies and cholestasis that provide therapeutic targets and genetic screening.^[25,28]^ Contribution of variant allele of the *ABCB11* gene encodes the BSEP, has been demonstrated to relate to susceptibility to intrahepatic cholestasis in different populations.^[20,24,29,30]^ Variants in the *ABCB11* gene are known associated with extrahepatic BA in Thailand patients,^[22]^ intrahepatic cholestasis in pregnancy,^[20,29]^ and early advanced fibrosis.^[24,31]^ However, to date, no studies have been performed to evaluate the role of this variant in the susceptibility to BA. Publications have been done in patients with cholestasis, especially in PFIC.^[19–21]^

In this study, we selected the patients who were confirmed with BA by Kasai surgery and liver biopsy for genotype analysis. We performed whole exome sequencing of several patients with BA to identify the variants that might be the cause of the disease. However, the variants that were identified in the *ABCC2, ERCC4, GPC1, ICAM1, ITGB2, MYO5B, NOTCH1, NOTCH2*, and *NOTCH3* genes in these patients (data not shown) are all benign in the ClinVar database. Besides, 2 gene variants (*ABCC2* rs927344 and *MYO5B* rs1815930) have been investigated to find the association with the disease in the BA patients.^[32]^ The variant in the *ABCC2* gene has been identified as a factor of the increasing bilirubin in the blood and a risk factor for cholestasis in pregnancy.^[33]^ The variants in the *MYO5B* gene have been reported as related to biliary anomalies leading to clinical symptoms of BA such as jaundice and intrahepatic cholestasis.^[34]^*MYO5B* mutations were reported to affect hepatic biliary function, increase the serum bile acid levels, and lead to cholestasis in the patients with or without MVID (microvillous inclusion disease).^[35–37]^ However, the results of genetic analysis (in the *ABCC2* and *MYO5B* genes) in Vietnamese patients showed no correlation between these variants and BA.^[32]^ Otherwise, the results of variant analysis in the *ABCB11* gene revealed that CC and TC genotypes were significantly different between BA patients and healthy people (*P* *<* *.01*), and the C allele was associated with an increased risk of BA (OR = 2.47; 95% CI: 1.84–3.32; *P* *<* *.01*). The frequency of TC + CC genotype of variant p.Val444Ala (c.1331T>C, rs2287622) in the *ABCB11* gene was significantly different in Vietnamese patients with BA disease. Although there has been no clear evidence linking this variant with susceptibility to BA, the results of genetic analysis about the genetic correlation of *ABCB11* variant p.Val444Ala with BA raised the hypothesis that cholestasis may be the cause of severe inflammatory cholangiopathy in BA. This variant can closely related to cholestasis and lead to serious damage to the biliary tract that obstructs the biliary tract in the patients. The results revealed an association between the *ABCB11* variant p.Val444Ala and the BA disease risk in Vietnamese patients.

In conclusion, the frequency variant p.Val444Ala in the *ABCB11* gene suggested that the C allele may be related to an increased risk of BA. Our result contributed to the general understanding of the cause of the disease and provided new insight into the role of this variant in the pathogenesis of BA.

## Acknowledgments

We are grateful to all patients who participated in this study.

## Author contributions

**Conceptualization:** Nguyen Thi Kim Lien, Nguyen Pham Anh Hoa.

**Data curation:** Nguyen Thi Phuong Mai, Pham Thi Hai Yen, Nguyen Pham Anh Hoa.

**Formal analysis:** Nguyen Thi Kim Lien, Nguyen Ngoc Lan.

**Funding acquisition:** Nguyen Huy Hoang.

**Investigation:** Nguyen Van Tung, Nguyen Ngoc Lan, Nguyen Thi Phuong Mai, Pham Thi Hai Yen.

**Methodology:** Nguyen Van Tung.

**Software:** Nguyen Van Tung.

**Writing – original draft:** Nguyen Van Tung, Nguyen Thi Kim Lien.

**Writing – review & editing:** Nguyen Thi Kim Lien, Nguyen Pham Anh Hoa, Nguyen Huy Hoang.
